# COVID-19: The Need for Immunoprevention at Industrial Scale

**DOI:** 10.4269/ajtmh.20-0239

**Published:** 2020-04-08

**Authors:** Scott B. Halstead

**Affiliations:** Consultant, North Bethesda, Maryland

Severe acute respiratory syndrome-CoV-2 infections are exacting devastating mortality in elderly persons with preexisting health conditions. This has led to widespread quarantining, suspension of social and business activities, frightening levels of morbidity and mortality, and enormous economic loss. As in the past, the nation has quickly found technical solutions to cope with this challenge. Industry has rapidly provided improved tests to detect viral RNA, respirator masks to prevent infections, and ventilators for critical intensive care; however, due to the scale of the pandemic, large needs remain. These and other requirements identified by the U.S. coronavirus task force have come from rapid and generous responses by industry.

Can we prevent these deaths? Vaccines, antiviral drugs, and antibodies are our tools of choice.

The best option would be a single-dose vaccine. Efforts to develop vaccines against Coronavirus disease-19 (COVID-19) are well advanced, based in part on experience with severe acute respiratory syndrome (SARS) and Middle East respiratory syndrome (MERS).^1^ However, before release, vaccines must demonstrate relevant immune responses in human volunteers, absence of unwanted side effects, and protection of humans from disease. These steps take many months to complete.

Antivirals have already achieved wide attention. A near-term solution is to repurpose existing drugs. Remdesivir, a nucleotide analog, is under clinical investigation in China and elsewhere. This drug has shown efficacy against MERS disease in a monkey model.^2^ Antibodies from convalescents or monoclonal antibodies derived from immunes, given early in illness, may reduce disease severity and save lives.^3^ Convalescent SARS antibodies given early in illness have been shown to reduce disease severity.^4^ In addition, it is likely that entirely new useful compounds will emerge from the laboratory.

Prevention of COVID-19 may be possible using antibodies. Previously in the United States, commercial gamma globulin provided short-term prevention against measles, paralytic poliomyelitis, and hepatitis A.^5–8^ In the 1950s, William McD Hammon at the University of Pittsburgh demonstrated that gamma globulin given to 100,000 children in a blinded efficacy trial successfully blunted paralysis attack rates during epidemics of poliomyelitis.^7^ Gamma globulin prepared from immune donors or protective monoclonal antibodies offer possibilities of short-term protection for care givers and healthcare workers and, in particular, for those at high risk of severe or fatal COVID-19. This latter group can be well identified by carefully designed epidemiological studies. Potential products must demonstrate protection in animal models and in small clinical trials. To avoid possible enhancement of COVID-19, antibodies might be given to prevent SARS-CoV-2 infections after the removal or inactivation of the IgG Fc terminus.^9^ Immunoprotection is an especially forceful solution that could be available soon. Efforts are well underway by many groups to derive monoclonal antibodies or manufacture gamma globulin from the huge cohort of COVID-19 convalescent immunes.^3,10^

Moving new products on line for early and effective use requires commitment and leadership. As suggested by Safi Bahcall (Wall Street Journal, March 21, 2020), a national science leader should immediately be appointed to organize an initial intervention, the direct immune protection of at-risk persons at industrial scale.
